# Supplementary material for: Exploiting volumetric wave correlation for enhanced depth imaging in scattering medium
Source: Nat Commun. 2023 Apr 4;14:1878. doi: 10.1038/s41467-023-37467-z (PMC10073116; doi:10.1038/s41467-023-37467-z)
Supplement: Supplementary file 1 — Supplementary Information [file 41467_2023_37467_MOESM1_ESM.pdf]

Supplementary Information

Exploiting volumetric wave correlation for enhanced depth imaging in scattering medium

Ye-Ryoung Lee[1,2,3,4,+], Dong-Young Kim[1,2,+], Yonghyeon Jo[1,2], Moonseok Kim[5,6], and Wonshik Choi[1,2,*]

[1]*Center for Molecular Spectroscopy and Dynamics, Institute for Basic Science, Seoul 02841, Korea*
[2]*Department of Physics, Korea University, Seoul 02841, Korea*
[3]*Institute of Basic Science, Korea University, Seoul 02841, Korea*
[4]*Department of Physics, Konkuk University, Seoul 05029, South Korea*
[5]*Department of Medical Life Sciences, College of Medicine, The Catholic University of Korea, Seoul, 06591, Korea*
[6]*Department of Biomedicine & Health Sciences, College of Medicine, The Catholic University of Korea, Seoul, 06591, Korea*
[+]These *authors contributed equally to this work*
[*]*wonshik@korea.ac.kr*

I. Experimental setup

We used two volumetric matrix measurement systems throughout our study, one with a supercontinuum laser and the other with a swept-source laser, depending on the types of applications. The details of the respective systems are introduced in the following.

1. Experimental schematic using a supercontinuum laser

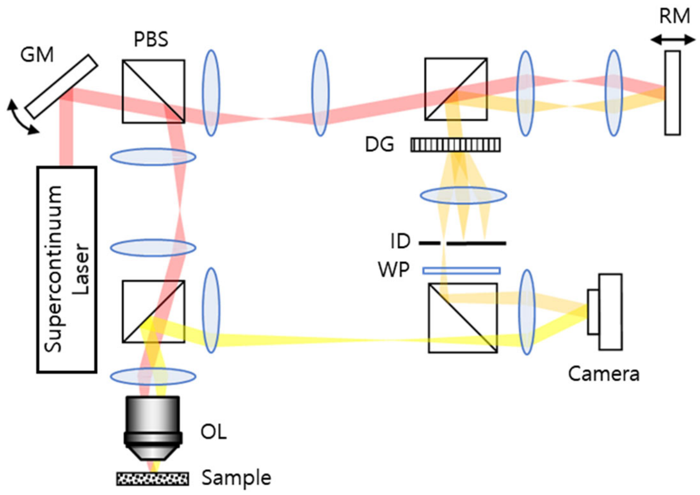

Figure S1. Detailed experimental setup using a supercontinuum laser. GM: a galvanometer scanning mirror, PBS: a polarizing beam splitter, OL: the objective lens, RM: the reference mirror, DG: the diffraction grating, ID: the iris diaphragm, WP: A wave plate.

The detailed layout of the experimental setup is shown in Fig. S1. A supercontinuum laser (NKT Photonics, model EXR-15) was used for scanning the wavelength of the light source. The center wavelength and bandwidth of the laser were tunable. The wavelength was scanned from 535 nm to 760 nm at an interval of 7.5 nm such that the number of sampling wavelengths was $N_\lambda = 29$. The estimated depth resolution was about 0.83 µm (air) from the spectral range of the laser. The bandwidth of the light source for each wavelength was $\Delta\lambda_S = 15\ nm$, whose coherence length was 12.4 µm. To match the temporal pulse front and the wavefront of sample and reference beams, the incident beam was scanned using a galvanometer mirror (GM, Cambridge Technology 6220H) and then divided into a sample beam and a reference beam at a polarizing beam splitter (PBS). The sample beam reflected off the PBS was sent to the objective lens (OL, 60X Nikon CFI Apochromat Objective 1.0 NA 2.8 mm WD) to illuminate the sample. The angle of galvanometer mirrors was scanned to uniformly cover the numerical aperture of the objective lens (60x Nikon CFI Apochromat, 1.0 NA, 2.8mm WD), and the number of the scanned incident angle was 2400. The backscattered wave from the sample was captured by the OL and sent to the camera (PCO. edge rolling shutter 4.2). The sample beam was magnified by a factor of 120 via the relay optics to the camera plane. The reference beam transmitted through the PBS was reflected from the reference mirror (RM), which was mounted on the translation stage to adjust the optical path length for temporal gating of interferometric detection. It was then sent to the diffraction grating (DG, Edmund Optics, 120 lp/mm). Its first-order diffraction was selected by the iris diaphragm (ID) and relayed to the camera to form an interferogram. A wave plate (WP) was used to set the polarization of the reference beam to be the same as that of the sample beam.

2. Experimental schematic using a swept-source laser

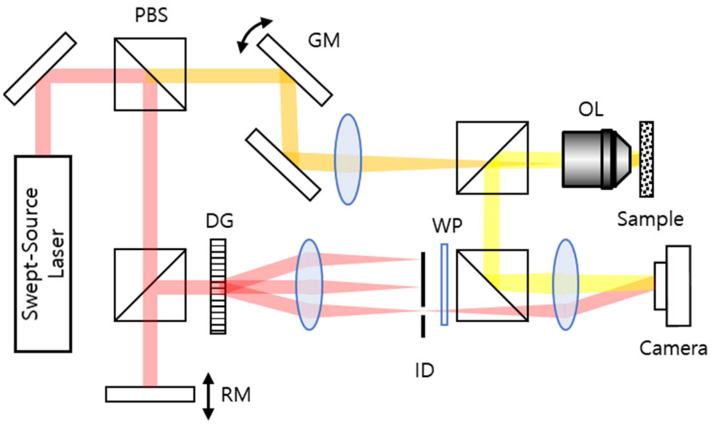

Figure S2. Detailed experimental setup using a swept-source laser. GM: a galvanometer scanning mirror, PBS: a polarizing beam splitter, OL: the objective lens, RM: the reference mirror, DG: the diffraction grating, ID: the iris diaphragm, WP: A wave plate.

The detailed layout of the experimental setup is shown in Fig. S2. We used a swept-source laser (Superlum BS-840-1-HP, spectral range: 803 nm ~ 878 nm). The wavelength was scanned from 803 nm to 878 nm at an interval of 1.25 nm such that the number of sampling wavelengths was $N_\lambda = 60$. The estimated depth resolution was about 4.2 μm (air) from the spectral range of the laser. The output beam from the laser was divided into a sample beam and a reference beam at a polarizing beam splitter (PBS). The sample beam transmitted through the PBS was sent to a galvanometer scanning mirror (GM, Thorlabs GVS011) for scanning the angle of illumination, and then the objective lens (OL, 60X Nikon CFI Apochromat Objective 1.0 NA 2.8 mm WD) to illuminate the sample. The angle of galvanometer mirrors was scanned to cover the targeted numerical aperture of the objective lens uniformly. For the progressive depth imaging experiments, the targeted numerical aperture was 0.8 NA at 542.5 nm, and it was 0.4 NA for the volumetric dispersion correction experiments. The number of the scanned incident angle was 1245 for both cases. The backscattered wave from the sample was captured by the OL and sent to the camera (PCO. edge rolling shutter 4.2). The sample beam was magnified by a factor of 60 via the relay optics to the camera plane. The reference beam reflected off the PBS was reflected from the reference mirror (RM) mounted on the translation stage to adjust the optical path length. It was then sent to the diffraction grating (DG, Edmund Optics, 72 lp/mm). Its first-order diffraction was selected by the iris diaphragm (ID) and relayed to the camera to form an interferogram with the sample beam. A wave plate (WP) was used to set the polarization of the reference beam to be the same as that of the sample beam.

Our system presents the first recording of a volumetric reflection matrix $\tilde{E}(\mathbf{k}; \mathbf{k}_{\text{in}}, \lambda)$, which is a measure of wide-field complex field maps in their output transverse wavevectors ($\mathbf{k}$) for the scanning of individual wavelengths (λ) and illumination angles ($\boldsymbol{\theta}_{\text{in}}$). It covers the best possible degrees of freedom in the context of elastic scattering. Any existing imaging modalities lack at least one or two degrees of freedom. For details, please see the following table.

<table>
<tr><th></th><th colspan="2">Wavelength</th><th colspan="2">Illumination angle</th><th colspan="2">Detection angle</th></tr>
<tr><th></th><th>integrated</th><th>resolved</th><th>integrated</th><th>resolved</th><th>integrated</th><th>resolved</th></tr>
<tr><td>TD-OCT</td><td>✓</td><td></td><td>✓</td><td></td><td>✓</td><td></td></tr>
<tr><td>FD/SS-OCT</td><td></td><td>✓</td><td>✓</td><td></td><td>✓</td><td></td></tr>
<tr><td>TD-FF-OCT</td><td>✓</td><td></td><td colspan="2">x</td><td></td><td>✓</td></tr>
<tr><td>SS-FF-OCT</td><td></td><td>✓</td><td colspan="2">x</td><td></td><td>✓</td></tr>
<tr><td>OCM</td><td>✓</td><td></td><td>✓</td><td></td><td>✓</td><td></td></tr>
<tr><td>BRM</td><td>✓</td><td></td><td></td><td>✓</td><td></td><td>✓</td></tr>
<tr><td>VRM</td><td></td><td>✓</td><td></td><td>✓</td><td></td><td>✓</td></tr>
</table>

Supplementary Table 1. Coverage of individual illumination/detection angles and wavelengths of various modalities. TD-OCT: time-domain optical coherence tomography (OCT). FD/SS-OCT: frequency-domain/swept-source OCT. TD-FF-OCT: time-domain full-field OCT. SS-FF-OCT: swept-source full-field OCT. OCM: optical coherence microscopy. BRM: broadband reflection matrix. VRM: volumetric reflection matrix.

II. Single-scattered waves from a volumetric object

For an incident wave $E_{\text{in}}(x,y,z) = e^{i\mathbf{k}_{\text{in}}\cdot\mathbf{r}+ik_z^{\text{in}}z} = e^{i(k_x^{\text{in}}x+k_y^{\text{in}}y+k_z^{\text{in}}z)}$ with the transverse and axial wavevectors $\mathbf{k}_{\text{in}} = (k_x^{\text{in}}, k_y^{\text{in}})$ and $k_z^{\text{in}} = \sqrt{k_0(\lambda)^2 - |\mathbf{k}_{\text{in}}|^2}$, respectively, let us consider that the detector plane is conjugate to $z = 0$ and the object function of a 2D section of a 3D object at $z = z_1(\neq 0)$ is $O(x,y,z_1)$. The incident wave at the position of the object is given by $E_{\text{in}}(x,y,z_1) = e^{i(\mathbf{k}_{\text{in}}\cdot\mathbf{r}+k_z^{\text{in}}z_1)}$, which is then reflected by the object. The reflected wave by the object at $z = z_1$ is given by $E(x,y,z_1;\mathbf{k}_{\text{in}}) = E_{\text{in}}(x,y,z_1) \times O(x,y,z_1)$. (Note: coordinate system in the reflection beam path is set such that x and y coordinates are the same as the incident beam path, and only z coordinate is reversed. This is to maintain transverse momentum after the reflection.)

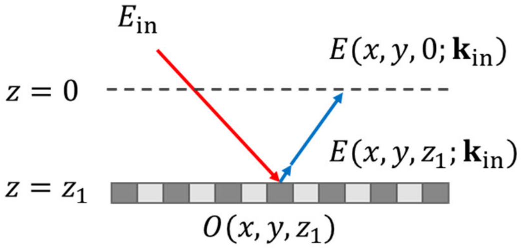

Figure S3. Single-scattered waves from a 2D section of a 3D object at $z = z_1$.

The angular spectrum of $E(x,y,z_1)$ is given by its Fourier transform with respect to x and y:

$$\begin{aligned}
\tilde{E}(k_x,k_y,z_1;\mathbf{k}_{\text{in}}) &= \iint E(x,y,z_1)e^{-i(k_xx+k_yy)}dxdy \\
&= \iint e^{i(k_x^{\text{in}}x+k_y^{\text{in}}y+k_z^{\text{in}}z_1)}\,O(x,y,z_1)e^{-i(k_xx+k_yy)}dxdy \\
&= \tilde{O}(k_x-k_x^{\text{in}},k_y-k_y^{\text{in}},z_1)e^{ik_z^{\text{in}}z_1} \\
&= \tilde{O}(\mathbf{k}-\mathbf{k}_{\text{in}},z_1)e^{ik_z^{\text{in}}z_1}.
\end{aligned} \tag{S1}$$

Since the detector plane is located at a conjugate to $z = 0$ plane, we need to find out the reflected wave $E(x,y,z=0;\mathbf{k}_{\text{in}})$ from $E(x,y,z_1;\mathbf{k}_{\text{in}})$ in its return path. This requires the free space propagation of $E(x,y,z_1;\mathbf{k}_{\text{in}})$ to $E(x,y,0;\mathbf{k}_{\text{in}})$ in the positive z-direction by the amount of $z = z_1$. Therefore, the angular spectrum of the reflected wave at $z = 0$ is given by

$$\begin{aligned}
\tilde{E}(\mathbf{k},z=0;\mathbf{k}_{\text{in}}) &= \tilde{E}(\mathbf{k},z_1;\mathbf{k}_{\text{in}})e^{ik_zz_1} \\
&= \tilde{O}(\mathbf{k}-\mathbf{k}_{\text{in}},z_1)e^{ik_z^{in}z_1}e^{ik_zz_1}.
\end{aligned} \tag{S2}$$

Here $k_z = \sqrt{k_0(\lambda)^2 - |\mathbf{k}|^2}$. For a general volumetric object composed of multiple layers along z, reflected wave measured at $z = 0$ is given by

$$\tilde{E}_{\text{r}}(\mathbf{k},z=0;\mathbf{k}_{\text{in}}) = \int \tilde{O}(\mathbf{k}-\mathbf{k}_{\text{in}},z)e^{ik_z^{in}z}e^{ik_zz}\,dz. \tag{S3}$$

Here $\tilde{O}(\mathbf{k},z)$ is the object spectrum at each depth with $\mathbf{k}$ the transverse wavevector.

III. Spectro-angular dispersion correction algorithm

Here, we elaborate on how to find the spectro-angular dispersion. As introduced in the main text, the single-scattered wave in the measured electric field $\tilde{E}(\mathbf{k};\mathbf{k}_{\text{in}},\lambda)$ is modified by the input and output phase retardations as

$$\tilde{E}_{\text{S}}(\mathbf{k};\mathbf{k}_{\text{in}},\lambda)=e^{i\phi_{\text{in}}(\mathbf{k}_{\text{in}},\lambda)}e^{i\phi_{\text{o}}(\mathbf{k},\lambda)}\int\gamma(z)\tilde{O}(\mathbf{k}-\mathbf{k}_{\text{in}},z)e^{ik_{z}^{\text{in}}(\lambda)z}\,e^{ik_{z}(\lambda)z}\,dz. \quad \text{(S4)}$$

The spectro-angular dispersion can be expressed as the sum of the angular dispersion of the center wavelength λ_{c} and the relative spectral dispersion with respect to λ_{c} for each $\mathbf{k}_{\text{in}}$ and $\mathbf{k}$:

$$\phi_{\text{in}}(\mathbf{k}_{\text{in}},\lambda)=\phi_{\text{in}}(\mathbf{k}_{\text{in}},\lambda_{\text{c}})+\Delta\phi_{\text{in}}(\mathbf{k}_{\text{in}},\lambda) \quad \text{(S5)}$$

$$\phi_{\text{o}}(\mathbf{k},\lambda)=\phi_{\text{o}}(\mathbf{k},\lambda_{\text{c}})+\Delta\phi_{\text{o}}(\mathbf{k},\lambda). \quad \text{(S6)}$$

Our spectro-angular dispersion correction algorithm first finds the spectral dispersions ($\Delta\phi_{\text{in}}(\mathbf{k}_{\text{in}},\lambda)$ and $\Delta\phi_{\text{o}}(\mathbf{k},\lambda)$) relative to the center wavelength and then find the angular dispersions ($\phi_{\text{in}}(\mathbf{k}_{\text{in}},\lambda_{\text{c}})$ and $\phi_{\text{o}}(\mathbf{k},\lambda_{\text{c}})$).

1. Correction of the spectral dispersion

First, the spectral dispersion for a fixed input wavevector can be obtained by calculating the correlation between output fields of different wavelengths with a fixed $\mathbf{k}_{\text{in}}$.

$$\begin{aligned}
&\langle\tilde{E}(\mathbf{k};\mathbf{k}_{\text{in}},\lambda)\tilde{E}^{*}(\mathbf{k};\mathbf{k}_{\text{in}},\lambda_{\text{c}})\rangle_{\mathbf{k}}\\
&=e^{i\Delta\phi_{\text{in}}(\mathbf{k}_{\text{in}},\lambda)}\langle e^{i\Delta\phi_{\text{o}}(\mathbf{k},\lambda)}\tilde{E}_{s}(\mathbf{k};\mathbf{k}_{\text{in}},\lambda)\tilde{E}_{s}{}^{*}(\mathbf{k};\mathbf{k}_{\text{in}},\lambda_{c})\rangle_{\mathbf{k}}\\
&+\langle\tilde{E}_{s}(\mathbf{k};\mathbf{k}_{\text{in}},\lambda)\tilde{E}_{M}{}^{*}(\mathbf{k};\mathbf{k}_{\text{in}},\lambda_{c})\rangle_{\mathbf{k}}+\langle\tilde{E}_{M}(\mathbf{k};\mathbf{k}_{\text{in}},\lambda)\tilde{E}_{s}{}^{*}(\mathbf{k};\mathbf{k}_{\text{in}},\lambda_{c})\rangle_{\mathbf{k}}\\
&+\langle\tilde{E}_{M}(\mathbf{k};\mathbf{k}_{\text{in}},\lambda)\tilde{E}_{M}{}^{*}(\mathbf{k};\mathbf{k}_{\text{in}},\lambda_{c})\rangle_{\mathbf{k}}.
\end{aligned} \quad \text{(S7)}$$

The correlation yields $\Delta\phi_{\text{in}}(\mathbf{k}_{\text{in}},\lambda)$ with an error $\delta\phi_{\text{in}}^{(1)}$ composed of three factors. The first is the output spectral dispersion, $e^{i\Delta\phi_{\text{o}}(\mathbf{k},\lambda)}$ that is summed with respect to different $\mathbf{k}$. The second is the defocus phase retardation, $e^{ik_{z}^{\text{in}}(\lambda)z}\,e^{ik_{z}(\lambda)z}$. The object functions at the out-of-focus plane are modified by the defocus phase retardations, and this phase retardation depends on wavelength. Thus, this phase retardation causes an error. The last factor is the random phase from multiple-scattered waves contributing to the last three terms in Eq. (S7). For minimizing the defocus error, a numerical propagation to the depth with the maximum reflectance (z_{max}) is applied to the obtained field prior to the correlation estimation because the target object with the maximum reflectance contributes dominantly to the correlation.

In the next step, the obtained input spectral dispersion correction, $\Delta\phi_{\text{in}}(\mathbf{k}_{\text{in}},\lambda)+\delta\phi_{\text{in}}^{(1)}(\mathbf{k}_{\text{in}},\lambda)$, is applied, and the spectral dispersion for a fixed output wavevector $\Delta\phi_{\text{o}}(\mathbf{k},\lambda)$ is obtained by calculating the correlation between input fields of different wavelengths, $\langle\tilde{E}(\mathbf{k};\mathbf{k}_{\text{in}},\lambda)\tilde{E}^{*}(\mathbf{k};\mathbf{k}_{\text{in}},\lambda_{c})\rangle_{\mathbf{k}_{\text{in}}}$. The correlation yields $\Delta\phi_{\text{o}}(\mathbf{k},\lambda)$ with an error $\delta\phi_{\text{o}}^{(1)}$ also affected by input spectral dispersion, defocused

object functions of other depths, and multiple-scattered waves. Then, calculating the correlation between $\Delta\phi_{\mathrm{o}}(\mathbf{k},\lambda)+\delta\phi_{\mathrm{o}}^{(1)}(\mathbf{k},\lambda)$ corrected output fields of different wavelengths with the same $\mathbf{k}_{\mathrm{in}}$ results in reduced error $\delta\phi_{\mathrm{in}}^{(2)}(\mathbf{k}_{\mathrm{in}},\lambda)$, as output spectral dispersion is considerably corrected. Again, the same process is applied for finding $\Delta\phi_{\mathrm{o}}(\mathbf{k},\lambda)+\delta\phi_{\mathrm{o}}^{(2)}(\mathbf{k},\lambda)$. By iterating this process, we can minimize the error $\delta\phi_{\mathrm{in}}^{(N)}(\mathbf{k}_{\mathrm{in}},\lambda)$ and $\delta\phi_{\mathrm{o}}^{(N)}(\mathbf{k},\lambda)$ and eventually find the relative spectral dispersion $\Delta\phi_{\mathrm{in}}(\mathbf{k}_{\mathrm{in}},\lambda)$ and $\Delta\phi_{\mathrm{o}}(\mathbf{k},\lambda)$ for each input/output wavevector and wavelength.

2. Correction of the angular dispersion

A summation of single-scattered waves with the same input wavevector along different wavelengths after spectral dispersion correction ($\Delta\phi_{\mathrm{in}}(\mathbf{k}_{\mathrm{in}},\lambda)$ and $\Delta\phi_{\mathrm{o}}(\mathbf{k},\lambda)$) and numerical focusing to a target depth yields the coherence-gated electric field

$$\begin{aligned}\tilde{E}_{\mathrm{cg}}^{S}(\mathbf{k},z_{\mathrm{t}};\mathbf{k}_{\mathrm{in}}) &= e^{-\Delta i\phi_{\mathrm{o}}(\mathbf{k},\lambda)}e^{-\Delta i\phi_{\mathrm{in}}(\mathbf{k}_{\mathrm{in}},\lambda)}\sum_{\lambda}\tilde{E}_{S}(\mathbf{k};\mathbf{k}_{\mathrm{in}},\lambda)e^{-ik_z^{\mathrm{in}}(\lambda)z_t}\,e^{-ik_z(\lambda)z_t}\\ &= e^{i\phi_{\mathrm{o}}(\mathbf{k},\lambda_{\mathrm{c}})}e^{i\phi_{\mathrm{in}}(\mathbf{k}_{\mathrm{in}},\lambda_{\mathrm{c}})}\int\sum_{\lambda}e^{-2z_t/l_s}\tilde{O}(\mathbf{k}-\mathbf{k}_{\mathrm{in}},z)\,e^{ik_z^{\mathrm{in}}(z-z_t)}\,e^{ik_z(z-z_t)}\,dz\\ &= e^{i\phi_{\mathrm{o}}(\mathbf{k},\lambda_{\mathrm{c}})}e^{i\phi_{\mathrm{in}}(\mathbf{k}_{\mathrm{in}},\lambda_{\mathrm{c}})}e^{-2z_t/l_s}\tilde{O}(\mathbf{k}-\mathbf{k}_{\mathrm{in}},z_t)N_{\lambda}.\end{aligned} \tag{S8}$$

Therefore, only the angular dispersions $\phi_{\mathrm{in}}(\mathbf{k}_{\mathrm{in}},\lambda_c)$ and $\phi_{\mathrm{o}}(\mathbf{k},\lambda_c)$ are to be determined in $\tilde{E}_{\mathrm{cg}}^{S}(\mathbf{k},z_{\mathrm{t}};\mathbf{k}_{\mathrm{in}})$. They can be found by a similar correlation estimation. However, since the output spectrum is shifted by the incident wavevector, the shift needs to be compensated prior to the correlation estimation. The input angular dispersion can be found by calculating the correlation between the shifted output fields for all the possible pairs of $\mathbf{k}_{\mathrm{in}}^{(i)}$ and $\mathbf{k}_{\mathrm{in}}^{(j)}$:

$$\begin{aligned}&\langle\tilde{E}_{\mathrm{cg}}\left(\mathbf{K},z_t;\mathbf{k}_{\mathrm{in}}^{(i)}\right)\tilde{E}_{\mathrm{cg}}^{*}\left(\mathbf{K},z_t;\mathbf{k}_{\mathrm{in}}^{(j)}\right)\rangle_{\mathbf{K}}\\ &= e^{i[\phi_{\mathrm{in}}(\mathbf{k}_{\mathrm{in}}^{(i)},\lambda_{\mathrm{c}})-\phi_{\mathrm{in}}(\mathbf{k}_{\mathrm{in}}^{(j)},\lambda_{\mathrm{c}})]}\langle e^{i[\phi_{\mathrm{o}}(\mathbf{K}+\mathbf{k}_{\mathrm{in}}^{(i)},\lambda_{\mathrm{c}})-\phi_{\mathrm{o}}(\mathbf{K}+\mathbf{k}_{\mathrm{in}}^{(j)},\lambda_{\mathrm{c}})]}\tilde{E}_{\mathrm{cg}}^{S}\left(\mathbf{K},z_t;\mathbf{k}_{\mathrm{in}}^{(i)}\right)\tilde{E}_{\mathrm{cg}}^{S\,*}\left(\mathbf{K},z_t;\mathbf{k}_{\mathrm{in}}^{(j)}\right)\rangle_{\mathbf{K}}\\ &+\langle\tilde{E}_{\mathrm{cg}}^{S}\left(\mathbf{K},z_t;\mathbf{k}_{\mathrm{in}}^{(i)}\right)\tilde{E}_{\mathrm{cg}}^{M\,*}\left(\mathbf{K},z_t;\mathbf{k}_{\mathrm{in}}^{(j)}\right)\rangle_{\mathbf{K}}+\langle\tilde{E}_{\mathrm{cg}}^{M}\left(\mathbf{K},z_t;\mathbf{k}_{\mathrm{in}}^{(i)}\right)\tilde{E}_{\mathrm{cg}}^{S\,*}\left(\mathbf{K},z_t;\mathbf{k}_{\mathrm{in}}^{(j)}\right)\rangle_{\mathbf{K}}\\ &+\langle\tilde{E}_{\mathrm{cg}}^{M}\left(\mathbf{K},z_t;\mathbf{k}_{\mathrm{in}}^{(i)}\right)\tilde{E}_{\mathrm{cg}}^{M\,*}\left(\mathbf{K},z_t;\mathbf{k}_{\mathrm{in}}^{(j)}\right)\rangle_{\mathbf{K}}.\end{aligned} \tag{S9}$$

Here, $\mathbf{K}=\mathbf{k}-\mathbf{k}_{\mathrm{in}}$. The correlation yields $\phi_{\mathrm{in}}(\mathbf{k}_{\mathrm{in}},\lambda_c)$ with an error $\delta\phi_{\mathrm{in}}^{(1)}(\mathbf{k}_{\mathrm{in}},\lambda_c)$, and the error is composed of two factors. The first is the shifted output angular dispersion, $e^{i\left[\phi_{\mathrm{o}}\left(\mathbf{K}+\mathbf{k}_{\mathrm{in}}^{(i)},\lambda_c\right)-\phi_{\mathrm{o}}\left(\mathbf{K}+\mathbf{k}_{\mathrm{in}}^{(j)},\lambda_c\right)\right]}$ that is summed with respect to $\mathbf{K}$. The second is the random phase from multiple-scattered waves, the last three terms in the summation. Note that the defocus error does not exist in this correlation due to coherence gating. Likewise, $\phi_{\mathrm{o}}(\mathbf{k},\lambda_c)$ is obtained by calculating the correlation of the shifted input fields of different $\mathbf{k}$, $\langle\tilde{E}_{\mathrm{cg}}(\mathbf{k}^{(i)},z_{\mathrm{t}};\mathbf{K})\tilde{E}_{\mathrm{cg}}^{*}(\mathbf{k}^{(j)},z_{\mathrm{t}};\mathbf{K})\rangle_{\mathbf{K}}$, with an error $\delta\phi_{o}^{(1)}(\mathbf{k},\lambda_c)$. By iterating this

process, we can minimize the error $\delta\phi_{\mathrm{i}}^{(N)}$ and $\delta\phi_{\mathrm{o}}^{(N)}$ and eventually find the $\phi_{\mathrm{in}}(\mathbf{k}_{\mathrm{in}},\lambda_{\mathrm{c}})$ and $\phi_{\mathrm{o}}(\mathbf{k},\lambda_{\mathrm{c}})$ for each input/output wavevector. At the end, we obtain $\phi_{\mathrm{in}}(\mathbf{k}_{\mathrm{in}},\lambda) = \phi_{\mathrm{in}}(\mathbf{k}_{\mathrm{in}},\lambda_{\mathrm{c}}) + \Delta\phi_{\mathrm{in}}(\mathbf{k}_{\mathrm{in}},\lambda)$ and $\phi_{\mathrm{o}}(\mathbf{k},\lambda) = \phi_o(\mathbf{k},\lambda_{\mathrm{c}}) + \Delta\phi_o(\mathbf{k},\lambda)$ in full for each λ, $\mathbf{k}_{\mathrm{in}}$, and $\mathbf{k}$.

The full process is simplified above for providing an intuitive picture. In detail, the correlation is obtained between confocal and coherence-gated field, $\widetilde{E}_{\mathrm{ccg}}^{(n)}(\mathbf{K},z_t) = \sum_{|\mathbf{k}_{\mathrm{in}}|\le k_0\alpha} \widetilde{E}_{\mathrm{cg}}^{(n)}(\mathbf{K},z_t;\mathbf{k}_{\mathrm{in}})$ with the nth correction and $\widetilde{E}_{\mathrm{cg}}^{(n)}$ of each $\mathbf{k}_{\mathrm{in}}$ (or $\mathbf{k}$) for maximizing the cross-correlation of single-scattered waves. Here we define n[th] correction coherence-gated field, input and output dispersion as $\widetilde{E}_{\mathrm{cg}}^{(n)}(\mathbf{K},z_t;\mathbf{k}_{\mathrm{in}}) \equiv \widetilde{E}_{\mathrm{cg}}(\mathbf{K},z_t;\mathbf{k}_{\mathrm{in}})e^{-i\phi_{\mathrm{in}}^{(n)}(\mathbf{k}_{\mathrm{in}},\lambda_{\mathrm{c}})}e^{-i\phi_{\mathrm{o}}^{(n)}(\mathbf{K}+\mathbf{k}_{\mathrm{in}},\lambda_{\mathrm{c}})}$, $\phi_{\mathrm{in}}^{(n)}(\mathbf{k}_{\mathrm{in}},\lambda_{\mathrm{c}}) \equiv \phi_{\mathrm{in}}(\mathbf{k}_{\mathrm{in}},\lambda_{\mathrm{c}}) + \delta\phi_{\mathrm{in}}^{(n)}(\mathbf{k}_{\mathrm{in}},\lambda_{\mathrm{c}})$, and $\phi_{\mathrm{o}}^{(n)}(\mathbf{K}+\mathbf{k}_{\mathrm{in}},\lambda_{\mathrm{c}}) \equiv \phi_{\mathrm{o}}(\mathbf{K}+\mathbf{k}_{\mathrm{in}},\lambda_{\mathrm{c}}) + \delta\phi_o^{(n)}(\mathbf{K}+\mathbf{k}_{\mathrm{in}},\lambda_{\mathrm{c}})$, respectively. Correlation between $\widetilde{E}_{\mathrm{ccg}}^{(n)}(\mathbf{K},z_t)$ and $\widetilde{E}_{\mathrm{cg}}^{(n)}\left(\mathbf{K},z_t;\mathbf{k}_{\mathrm{in}}^{(j)}\right)$ provides better fidelity in comparison with the correction between individual coherence-gated fields given in Eq. (S9).

3. Object function reconstruction after spectro-angular dispersion correction

Once the spectro-angular dispersions are identified, we compensate the $\phi_{\mathrm{in}}(\mathbf{k}_{\mathrm{in}},\lambda)$ and $\phi_{\mathrm{o}}(\mathbf{k},\lambda)$ for the measured field, $\tilde{E}(\mathbf{k};\mathbf{k}_{\mathrm{in}},\lambda)$, and apply a proper numerical propagation to the desired depth (z_t) by multiplying $e^{-ik_z^{\mathrm{in}}(\lambda)z_t}\, e^{-ik_z(\lambda)z_t}$:

$$\begin{aligned}\tilde{E}_{\mathrm{c}}(\mathbf{k};\mathbf{k}_{\mathrm{in}},\lambda) &= \tilde{E}(\mathbf{k};\mathbf{k}_{\mathrm{in}},\lambda)e^{-i\phi_{\mathrm{in}}(\mathbf{k}_{\mathrm{in}},\lambda)}e^{-i\phi_{\mathrm{o}}(\mathbf{k},\lambda)}e^{-ik_z^{\mathrm{in}}(\lambda)z_t}\, e^{-ik_z(\lambda)z_t} \\ &= \int e^{-2z/l_s}\tilde{O}(\mathbf{k}-\mathbf{k}_{\mathrm{in}},z)e^{ik_z^{\mathrm{in}}(\lambda)(z-z_t)}\, e^{ik_z(\lambda)(z-z_t)}\, dz \\ &\quad + \tilde{E}_{\mathrm{M}}(\mathbf{k};\mathbf{k}_{\mathrm{in}},\lambda)e^{-i\phi_{\mathrm{in}}(\mathbf{k}_{\mathrm{in}},\lambda)}e^{-i\phi_{\mathrm{o}}(\mathbf{k},\lambda)}e^{-ik_z^{\mathrm{in}}(\lambda)z_t}\, e^{-ik_z(\lambda)z_t}.\end{aligned} \tag{S10}$$

Coherent addition of $\tilde{E}_{\mathrm{c}}(\mathbf{k};\mathbf{k}_{\mathrm{in}},\lambda)$ with respect to λ yields $e^{-2z/l_s}\tilde{O}(\mathbf{k}-\mathbf{k}_{\mathrm{in}},z)N_\lambda$

$$\begin{aligned}\tilde{E}_{\mathrm{cg}}(\mathbf{k},z_t;\mathbf{k}_{\mathrm{in}},\lambda) &= \sum_\lambda \tilde{E}_{\mathrm{c}}(\mathbf{k};\mathbf{k}_{\mathrm{in}},\lambda) \\ &\approx e^{-2z_t/l_s}\tilde{O}(\mathbf{k}-\mathbf{k}_{\mathrm{in}},z_t)N_\lambda + \sum_\lambda \tilde{E}_{\mathrm{M}}'(\mathbf{k};\mathbf{k}_{\mathrm{in}},\lambda).\end{aligned} \tag{S11}$$

Here we assume that summation of $e^{ik_z^{\mathrm{in}}(\lambda)(z-z_t)}\, e^{ik_z(\lambda)(z-z_t)}$ with respect to λ disappears when $z \neq z_t$. The multiple-scattered waves after the dispersion and defocus compensations, $\tilde{E}_{\mathrm{M}}'(\mathbf{k};\mathbf{k}_{\mathrm{in}},\lambda)$, remains similar to $\tilde{E}_{\mathrm{M}}(\mathbf{k};\mathbf{k}_{\mathrm{in}},\lambda)$ in their wave correlation properties. We then perform the summation of $\tilde{E}_{\mathrm{cg}}(\mathbf{k};\mathbf{k}_{\mathrm{in}},\lambda)$ with respect to $\mathbf{k}_{\mathrm{in}}$ after the spectral shifts ($\mathbf{K} = \mathbf{k} - \mathbf{k}_{\mathrm{in}}$)

$$
\begin{aligned}
&\tilde{E}_{\mathrm{ccg}}(\mathbf{K}, z_t) \\
&= \sum_{|\boldsymbol{k}_{\mathrm{in}}| \le k_0 \alpha} \tilde{E}_{\mathrm{cg}}(\mathbf{K}, z_t; \mathbf{k}_{\mathrm{in}}) \\
&\approx \gamma(z) \tilde{O}(\mathbf{K}, z_t) N_\lambda N(\mathbf{K}) + \sum_{|\boldsymbol{k}_{\mathrm{in}}| \le k_0 \alpha} \sum_{\lambda} \tilde{E}_{\mathrm{M}}(\mathbf{k}; \mathbf{k}_{\mathrm{in}}, \lambda)
\end{aligned}
\tag{S12}
$$

Here $N(\mathbf{K})$ is the number of $\mathbf{k}_{\mathrm{in}}$ and $\mathbf{k}$ pairs that meet the relation, $\mathbf{K} = \mathbf{k} - \mathbf{k}_{\mathrm{in}}$. The magnitude of the multiple scattering term is given approximately as $|\tilde{E}_{\mathrm{M}}(\mathbf{k}; \mathbf{k}_{\mathrm{in}}, \lambda)|\sqrt{N_\lambda N(\mathbf{K})}$ when the multiple scattering is fully decorrelated. When $\gamma(z)\tilde{O}(\mathbf{k} - \mathbf{k}_{\mathrm{in}}, z_t) N_\lambda N(\mathbf{K}) \gg |\tilde{E}_{\mathrm{M}}(\mathbf{k}; \mathbf{k}_{\mathrm{in}}, \lambda)|\sqrt{N_\lambda N(\mathbf{K})}$, then $\tilde{E}_{\mathrm{ccg}}(\mathbf{K}, z_t) \approx \gamma(z)\tilde{O}(\mathbf{K}, z_t) N_\lambda N(\mathbf{K})$. Essentially, we found $\tilde{O}(\mathbf{K}, z)$ for each $z = z_t$ from $\tilde{E}_{\mathrm{c}}(\mathbf{k}; \mathbf{k}_{\mathrm{in}}, \lambda)$.

IV. Characterization of scattering media in terms of PSF attenuation

The scattering media used in our experiments induce both multiple scattering and spectro-angular dispersions. These kinds of hybrid complex media are common in biological tissues, and brain tissue under a skull is a good example. Here we first introduce a way to characterize the hybrid complex medium in the context of high-resolution imaging exploiting ballistic waves over a wide range of wavevectors. Essentially, the scattering mean free path and Strehl ratio are the parameters defining the difficulty in the high-resolution imaging of an embedded object (Fig. S4).

1. Scattering mean free path (Fig. S4a): The ballistic component of each planar wave constituting a focused illumination is attenuated exponentially in its intensity with the depth. The ballistic wave attenuation is given by $\exp(-z/l_{\mathrm{s}})$ with l_{s} the scattering mean free path. Multiple-scattered waves generated in this process serve as background. As a result, the peak intensity of the point-spread-function (PSF) formed by the ballistic waves is attenuated by the same factor. This attenuation is not recoverable unless there is a strategy to make the deterministic use of multiple scattering.

2. Strehl ratio (Fig. S4b): Angle- and wavelength-dependent phase retardations by the complex medium further attenuate the peak intensity of the PSF by hampering the constructive interference of the ballistic waves in forming a focused spot. Strehl ratio η is defined by the attenuation of the peak PSF intensity by the spectro-angular dispersions. The attenuation of PSF by the dispersions can be restored either computationally or experimentally by means of compensating the phase retardations.

Scattering mean free path is a good parameter for characterizing the effect of multiple scattering. However, in high-resolution imaging, we need to consider the coherent summation of waves with a wide range of wavevectors to ensure optimal confocal and coherence gating. The peak PSF intensity is attenuated by the joint action of the scattering and dispersions, $I(z)/I_0 = \eta \times exp(-z/l_s)$. To unify

the effects of scattering and aberration, we consider the PSF attenuation by the dispersions as an addition of a scattering layer with the effective thickness z_{eff} given by the following relation.

$$I(z_{\text{eff}}) = \eta \times I_0 = I_0 e^{-z_{\text{eff}}/l_s} \tag{S13}$$

$$z_{\text{eff}} = -\ln(\eta)\, l_s \tag{S14}$$

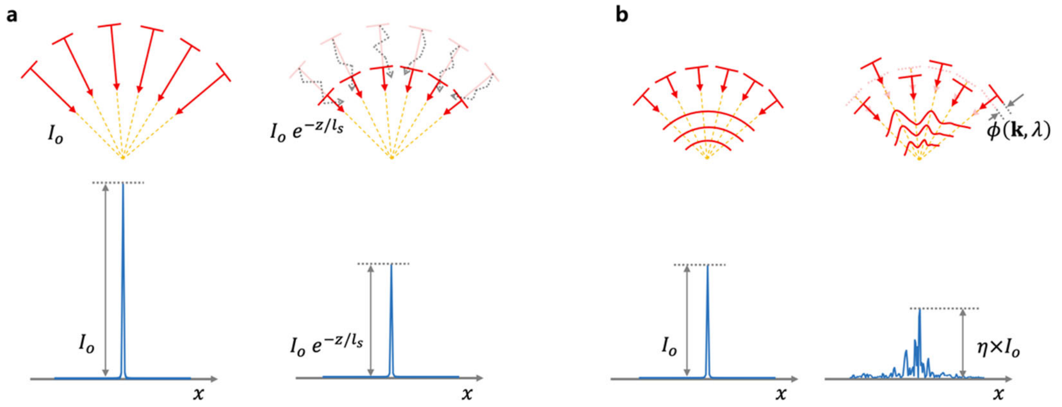

Figure S4. Multiple scattering and spectro-angular dispersion. a, Due to multiple scattering, the ballistic component of each planar wave constituting a focused illumination is attenuated by $exp(-z/l_s)$ with l_s the scattering mean free path. **b,** Angle- and wavelength-dependent phase retardations ($\phi(\mathbf{k}, \lambda)$) by the complex medium further attenuate the peak intensity of the PSF by hampering the constructive interference of the ballistic waves in forming a focus. Strehl ratio η is defined by the attenuation of the peak PSF intensity by the spectro-angular dispersions.

The Strehl ratios of the scattering media used in the experiments were estimated by the PSFs obtained from the spectro-angular dispersions. The measured Strehl ratios were 0.012 (Fig. 4), 0.011 (Fig. 5, upper layer), 0.004 (Fig. 5, lower layer), and 0.011 (Fig. 6). They correspond to adding additional scattering layers with the optical thicknesses of 4.4 l_s, 4.6 l_s, 5.6 l_s, and 4.6 l_s, respectively.

We determined the optical thickness of the scattering media in terms of scattering mean free paths by measuring the attenuation of planar waves with various illumination angles and wavelengths covered in our experiments. The thicknesses of the scattering media were $3.6 - 5.0\ l_s$ (Fig. 4), $1.4 - 1.7\ l_s$ (Fig. 5, upper layer) and $3.0 - 3.3\ l_s$ (Fig. 5, lower layer), and $1.8 - 2.3\ l_s$ (Fig. 6). Therefore, the total optical thicknesses of the hybrid scattering media used in experiments were $8.0 - 9.4\ l_s$ (Fig. 4), $6.0 - 6.3\ l_s$ (Fig. 5, upper layer), $8.6 - 8.9\ l_s$(Fig. 5, lower layer), and $6.4 - 6.9\ l_s$ (Fig. 6) in terms of the PSF attenuation in confocal imaging.

		Fig. 4	Fig. 5, z_U	Fig. 5, z_L	Fig. 6
Multiple scattering	Optical thickness [l_s]	3.6~5.0	1.4~1.7	3.0~3.3	1.8~2.3
Dispersion	Strehl ratio	0.012	0.011	0.004	0.011
	Effective optical thickness [l_s]	4.4	4.6	5.6	4.6
Total optical thickness [l_s]		8.0~9.4	6.0~6.3	8.6~8.9	6.4~6.9

Supplementary Table 2. Scattering properties of the samples used in the experiments. Since we used an ultra-broadband source, the optical thickness has a wavelength-dependent range.

On a separate note, the PSF attenuation occurs twice during the round trip, i.e. $(I(z)/I_0)^2 = \eta^2 \times exp(-2z/l_s)$, in the reflection-mode imaging of an embedded object, making the image reconstruction problems much worse. The scattering property of a complex medium is mainly characterized by the scattering and transport mean free paths. In the context of high-resolution imaging, the scattering mean free path is a suitable parameter as any momentum change disrupts imaging resolution. In the case of deterministic use of multiple scattering for image reconstruction, the transport mean free path can be a good parameter. However, such an implementation is yet to come in the context of deep imaging.

V. Siemens Star-like target

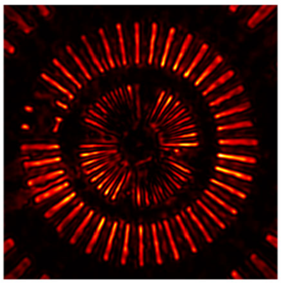

Figure S5. The image of the resolution target used for the experiments.

Fig. S5 shows the image of the Siemen star-like target used in the experiment obtained without aberrating/scattering medium above, which can be considered as the ground truth image. The reflectance of the target was rather uneven due to imperfect fabrication.

VI. Turbidity dependence on wavelengths

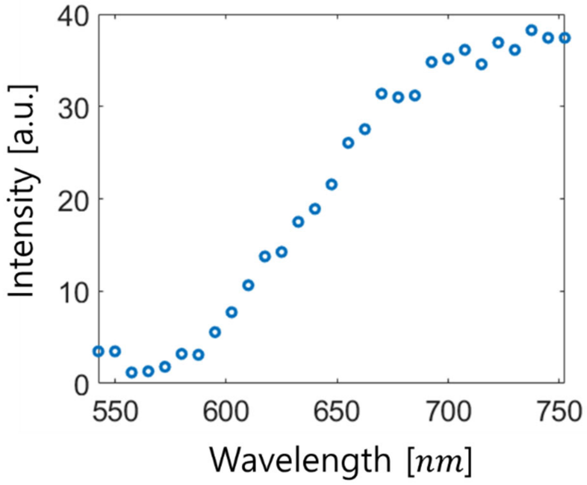

Figure S6. The signal intensity measured from VRM images versus center wavelengths when the total intensities of raw images are normalized.

Fig. S6 shows the signal intensity measured from VRM images for different center wavelengths when the total intensities of raw images were normalized. We used the spectral bandwidth coverage $\Delta\lambda_F$ of 30 nm because VRM images could not be reconstructed for $\Delta\lambda_F$ of 15 nm. For the center wavelength of 542.5 nm and 752.5 nm, we adopted the value of the nearest wavelength as a rough estimation.

VII. Dispersion correction condition

1. Theoretical derivation of the dispersion correction condition

The dispersion can be found by finding the phase of each input channel that maximizes the total intensity of single-scattered waves. This phase can be found by the correlation $\langle \tilde{E}_{\mathrm{ccg}}^{(n)}(\mathbf{K}, z_t)\, \tilde{E}_{\mathrm{cg}}^{*(n)}\left(\mathbf{K}+\mathbf{k}_{\mathrm{in}}^{(j)}, z_t; \mathbf{k}_{\mathrm{in}}^{(j)}\right)\rangle_{\mathbf{K}}$, where the correlation of single-scattered waves is hampered by the presence of multiple-scattered waves and the output (input) dispersion in the case of finding the input (output) dispersion.

The coherence-gated field after nth correction can be approximated as

$$
\begin{aligned}
\tilde{E}_{\mathrm{cg}}^{(n)}(\mathbf{k}, z_t; \mathbf{k}_{\mathrm{in}}) &= \sum_{\lambda} \tilde{E}(\mathbf{k}; \mathbf{k}_{\mathrm{in}}, \lambda) e^{-ik_z^{\mathrm{in}}(\lambda) z_t}\, e^{-ik_z(\lambda) z_t} e^{-i\phi_{\mathrm{in}}^{(n)}(\mathbf{k}_{\mathrm{in}}, \lambda_c)} e^{-i\phi_{\mathrm{o}}^{(n)}(\mathbf{k}, \lambda_c)} \\
&= \tilde{E}_{\mathrm{cg}}^{\mathrm{S}\,(n)}(\mathbf{k}, z_t; \mathbf{k}_{\mathrm{in}}) + \tilde{E}_{\mathrm{cg}}^{\mathrm{M}\,(n)}(\mathbf{k}, z_t; \mathbf{k}_{\mathrm{in}}) \\
&\approx e^{-i\delta\phi_{\mathrm{in}}^{(n)}(\mathbf{k}_{\mathrm{in}}, \lambda_c)} e^{-i\delta\phi_{\mathrm{o}}^{(n)}(\mathbf{k}, \lambda_c)} \gamma(z_t) \tilde{O}(\mathbf{k}-\mathbf{k}_{\mathrm{in}}, z_t) N_\lambda + \tilde{E}_{\mathrm{cg}}^{\mathrm{M}}(\mathbf{k}, z_t; \mathbf{k}_{\mathrm{in}}).
\end{aligned}
\qquad \text{(S14)}
$$

The correlation can be rewritten as follows:

$$\begin{aligned}
&\langle \tilde{E}_{\mathrm{ccg}}^{(n)}(\mathbf{K}, z_t)\, \tilde{E}_{\mathrm{cg}}^{*(n)}\left(\mathbf{K}, z_t; \mathbf{k}_{\mathrm{in}}^{(j)}\right)\rangle_{\mathbf{K}} \\
&\approx \sum_{\mathbf{k}_{\mathrm{in}}^{(i)}} e^{-i\left[\delta\phi_{\mathrm{in}}^{(n)}\left(\mathbf{k}_{\mathrm{in}}^{(i)},\lambda_{\mathrm{c}}\right)-\delta\phi_{\mathrm{in}}^{(n)}\left(\mathbf{k}_{\mathrm{in}}^{(j)},\lambda_{\mathrm{c}}\right)\right]} \langle e^{-i\left[\delta\phi_{\mathrm{o}}^{(n)}\left(\mathbf{K}+\mathbf{k}_{\mathrm{in}}^{(i)},\lambda_{\mathrm{c}}\right)-\delta\phi_{\mathrm{o}}^{(n)}\left(\mathbf{K}+\mathbf{k}_{\mathrm{in}}^{(j)},\lambda_{\mathrm{c}}\right)\right]} \left|\gamma(z_t) N_\lambda \tilde{O}(\mathbf{K}, z_t)\right|^2 \rangle_{\mathbf{K}} \\
&\qquad + \langle \sum_{\mathbf{k}_{\mathrm{in}}^{(i)}} \tilde{E}_{\mathrm{cg}}^{M\,(n)}\left(\mathbf{K}, z_t; \mathbf{k}_{\mathrm{in}}^{(i)}\right) \tilde{E}_{\mathrm{cg}}^{M\,(n)^*}\left(\mathbf{K}, z_t; \mathbf{k}_{\mathrm{in}}^{(j)}\right)\rangle_{\mathbf{K}} \\
&\approx \sum_{\mathbf{k}_{\mathrm{in}}^{(i)}} e^{-i\left[\delta\phi_{\mathrm{in}}^{(n)}\left(\mathbf{k}_{\mathrm{in}}^{(i)},\lambda_{\mathrm{c}}\right)-\delta\phi_{\mathrm{in}}^{(n)}\left(\mathbf{k}_{\mathrm{in}}^{(j)},\lambda_{\mathrm{c}}\right)\right]} \xi\left(\mathbf{k}_{\mathrm{in}}^{(i)}, \mathbf{k}_{\mathrm{in}}^{(j)}\right) N_{\mathbf{K}}\left(\mathbf{k}_{\mathrm{in}}^{(i)}, \mathbf{k}_{\mathrm{in}}^{(j)}\right) S + e^{i\phi_M^{(n)}} \sqrt{\sum_{\mathbf{k}_{\mathrm{in}}^{(i)}} N_{\mathbf{K}}\left(\mathbf{k}_{\mathrm{in}}^{(i)}, \mathbf{k}_{\mathrm{in}}^{(j)}\right)}\, M \\
&\approx e^{i\delta\phi_{\mathrm{in}}^{(n)}\left(\mathbf{k}_{\mathrm{in}}^{(j)},\lambda_{\mathrm{c}}\right)} S \sum_{\mathbf{k}_{\mathrm{in}}^{(i)}} e^{-i\delta\phi_{\mathrm{in}}^{(n)}\left(\mathbf{k}_{\mathrm{in}}^{(i)},\lambda_{\mathrm{c}}\right)} \xi\left(\mathbf{k}_{\mathrm{in}}^{(i)}, \mathbf{k}_{\mathrm{in}}^{(j)}\right) N_{\mathbf{K}}\left(\mathbf{k}_{\mathrm{in}}^{(i)}, \mathbf{k}_{\mathrm{in}}^{(j)}\right) + e^{i\phi_M^{(n)}} \sqrt{\sum_{\mathbf{k}_{\mathrm{in}}^{(i)}} N_{\mathbf{K}}\left(\mathbf{k}_{\mathrm{in}}^{(i)}, \mathbf{k}_{\mathrm{in}}^{(j)}\right)}\, M.
\end{aligned}$$

(S15)

Here S and M are ensemble averaged single and multiple scattering intensities at each independent output channel ($S = \frac{\sum_{\mathbf{k}_{\mathrm{in}}} \langle |\tilde{E}_{\mathrm{cg}}^{S}(\mathbf{K}+\mathbf{k}_{\mathrm{in}}, z_t; \mathbf{k}_{\mathrm{in}})|^2 \rangle_{\mathbf{K}} / N_{\mathbf{K}}}{N(\mathbf{k}_{\mathrm{in}})}$ and $M = \frac{\sum_{\mathbf{k}_{\mathrm{in}}} \langle |\tilde{E}_{\mathrm{cg}}^{M}(\mathbf{K}+\mathbf{k}_{\mathrm{in}}, z_t; \mathbf{k}_{\mathrm{in}})|^2 \rangle_{\mathbf{K}} / N_{\mathbf{K}}}{N(\mathbf{k}_{\mathrm{in}})}$), and S is mainly determined by $\langle \left|\gamma(z_t) N_\lambda \tilde{O}(\mathbf{K}, z_t)\right|^2 \rangle_{\mathbf{K}}$. $\xi\left(\mathbf{k}_{\mathrm{in}}^{(i)}, \mathbf{k}_{\mathrm{in}}^{(j)}\right)$ is a measure of the complexity of the output angular dispersion defined by $\xi\left(\mathbf{k}_{\mathrm{in}}^{(i)}, \mathbf{k}_{\mathrm{in}}^{(j)}\right) \equiv \langle e^{-i\left[\delta\phi_o^{(n)}\left(\mathbf{K}+\mathbf{k}_{\mathrm{in}}^{(i)},\lambda_c\right)-\delta\phi_o^{(n)}\left(\mathbf{K}+\mathbf{k}_{\mathrm{in}}^{(j)},\lambda_c\right)\right]}\rangle_{\mathbf{K}} / N_{\mathbf{K}}\left(\mathbf{k}_{\mathrm{in}}^{(i)}, \mathbf{k}_{\mathrm{in}}^{(j)}\right)$. The compensation of the output spectrum shift by the incident wavevector naturally shifts the pupil function for each input. $N_{\mathbf{K}}\left(\mathbf{k}_{\mathrm{in}}^{(i)}, \mathbf{k}_{\mathrm{in}}^{(j)}\right)$ is number of independent output channels within the overlapping pupil function of $\mathbf{k}_{\mathrm{in}}^{(i)}$ and $\mathbf{k}_{\mathrm{in}}^{(j)}$. Without the shift compensation, $N_{\mathbf{K}}\left(\mathbf{k}_{\mathrm{in}}^{(i)}, \mathbf{k}_{\mathrm{in}}^{(j)}\right)$ is simply number of independent output channels, N.

The correlation in Eq. (S15) can be explained as follows. First, the amplitude of the correlation is proportional to $N_{\mathbf{K}}\left(\mathbf{k}_{\mathrm{in}}^{(i)}, \mathbf{k}_{\mathrm{in}}^{(j)}\right)$ for single-scattered waves because they share the same phasor which is cancelled at the time of inner product. On the other hand, multiple-scattered wave correlation is proportional to $\sqrt{N_{\mathbf{K}}\left(\mathbf{k}_{\mathrm{in}}^{(i)}, \mathbf{k}_{\mathrm{in}}^{(j)}\right)}$ due to the addition of random phasors. Second, the output spectral dispersion attenuates the single scattering correlation since $\xi\left(\mathbf{k}_{\mathrm{in}}^{(i)}, \mathbf{k}_{\mathrm{in}}^{(j)}\right)$ is smaller than 1. Note that the output angular dispersion spoils the single scattering correlation because it is shifted differently depending on the incident wavevectors. The convergence of this correlation to the correct dispersion can be understood as competition between the correlation of single-scattered waves with an amplitude of $\zeta\left(\mathbf{k}_{\mathrm{in}}^{(j)}\right) \sum_{\mathbf{k}_{\mathrm{in}}^{(i)}} N_{\mathbf{K}}\left(\mathbf{k}_{\mathrm{in}}^{(i)}, \mathbf{k}_{\mathrm{in}}^{(j)}\right) S$ and that of multiple-scattered waves with an amplitude of

$\sqrt{\sum_{\mathbf{k}_{\text{in}}^{(i)}} N_{\mathbf{K}}\left(\mathbf{k}_{\text{in}}^{(i)}, \mathbf{k}_{\text{in}}^{(j)}\right)} M$, where $\zeta\left(\mathbf{k}_{\text{in}}^{(j)}\right) \equiv \frac{\left|\sum_{\mathbf{k}_{\text{in}}^{(i)}} e^{i\delta\phi_{\text{in}}^{(n)}\left(\mathbf{k}_{\text{in}}^{(i)}, \lambda_c\right)} \xi\left(\mathbf{k}_{\text{in}}^{(i)}, \mathbf{k}_{\text{in}}^{(j)}\right) N_{\mathbf{K}}\left(\mathbf{k}_{\text{in}}^{(i)}, \mathbf{k}_{\text{in}}^{(j)}\right)\right|}{\sum_{\mathbf{k}_{\text{in}}^{(i)}} N_{\mathbf{K}}\left(\mathbf{k}_{\text{in}}^{(i)}, \mathbf{k}_{\text{in}}^{(j)}\right)}$. In other words, the relative contribution of single-scattered waves and multiple-scattered waves to the cross-correlation, $C_{\text{rel}}\left(\mathbf{k}_{\text{in}}^{(j)}\right) = \frac{S}{M}\zeta\left(\mathbf{k}_{\text{in}}^{(j)}\right)\sqrt{\sum_{\mathbf{k}_{\text{in}}^{(i)}} N_{\mathbf{K}}\left(\mathbf{k}_{\text{in}}^{(i)}, \mathbf{k}_{\text{in}}^{(j)}\right)}$, needs to be larger than a finite threshold, the specific value of which depends on noise level.

Note that $\zeta\left(\mathbf{k}_{\text{in}}^{(j)}\right)$ indicates the complexity of both input and output angular dispersion, and $\zeta\left(\mathbf{k}_{\text{in}}^{(j)}\right) = 1$ when both input and output dispersions do not exist. $C_{\text{rel}}\left(\mathbf{k}_{\text{in}}^{(j)}\right)$ is maximum when $\mathbf{k}_{\text{in}}^{(j)} = 0$ because $N_{\mathbf{K}}\left(\mathbf{k}_{\text{in}}^{(i)}, \mathbf{k}_{\text{in}}^{(j)}\right)$ is maximum when $\mathbf{k}_{\text{in}}^{(j)} = 0$. Since $\zeta\left(\mathbf{k}_{\text{in}}^{(j)} = 0\right)$ reflects the overall complexity of the angular dispersion by covering most of the overlapping pupil function, we define $C_{\text{rel}} \equiv C_{\text{rel}}\left(\mathbf{k}_{\text{in}}^{(j)} = 0\right)$ for evaluation of dispersion correction condition such that

$$C_{\text{rel}} = \frac{S}{M}\zeta\sqrt{N}, \tag{S12}$$

where $\zeta \equiv \zeta\left(\mathbf{k}_{\text{in}}^{(j)} = 0\right)$ and $N \equiv \sum_{\mathbf{k}_{\text{in}}^{(i)}} N_{\mathbf{K}}\left(\mathbf{k}_{\text{in}}^{(i)}, \mathbf{k}_{\text{in}}^{(j)} = 0\right)$. N is interpreted as total number of elements involved in the cross-correlation of pupil functions.

2. Experimental validation of the derived condition

The working condition for dispersion correction is set by $C_{\text{rel}} = (S/M)\zeta\sqrt{N} \geq C_{\text{th}}$. Here, S and M are ensemble-averaged single and multiple scattering intensities at each independent output channel. N is approximately the number of independent output channels times the number of independent input channels, and ζ indicates the complexity of both input and output angular dispersions. We examined this condition after coherence gating. Therefore, S/M depends linearly on the bandwidth, or the number of wavelength channels, N_λ. N is proportional to the number of input angular channels N_θ for a given number of output channels. Essentially, C_{rel} is proportional to $N_\lambda\sqrt{N_\theta}$ such that the depth limit is set by $N_\lambda\sqrt{N_\theta} = constant$.

Figure R7 shows the image reconstruction results for various combinations of bandwidth and number of input channels. The conditions where the image reconstruction is successful are indicated in the green-shaded area, while those where the image reconstruction failed were indicated in the red-shaded area. The borderline between the two shaded areas approximately matches the contour set by $N_\lambda\sqrt{N_{in}} = constant$, as expected.

From this result, we could estimate the achievable imaging depth. When the bandwidth is 225 nm, the method works until N_θ decreases to 600 in Fig. S7. The total number of independent input angular channels of the system is approximately 5500 (NA=1.0 and field of view=22.8 μm x 22.8 μm at 542.5 nm). If we use the full number of input channels, the method will work even when S/M is further attenuated by $\sqrt{600/5500}$.

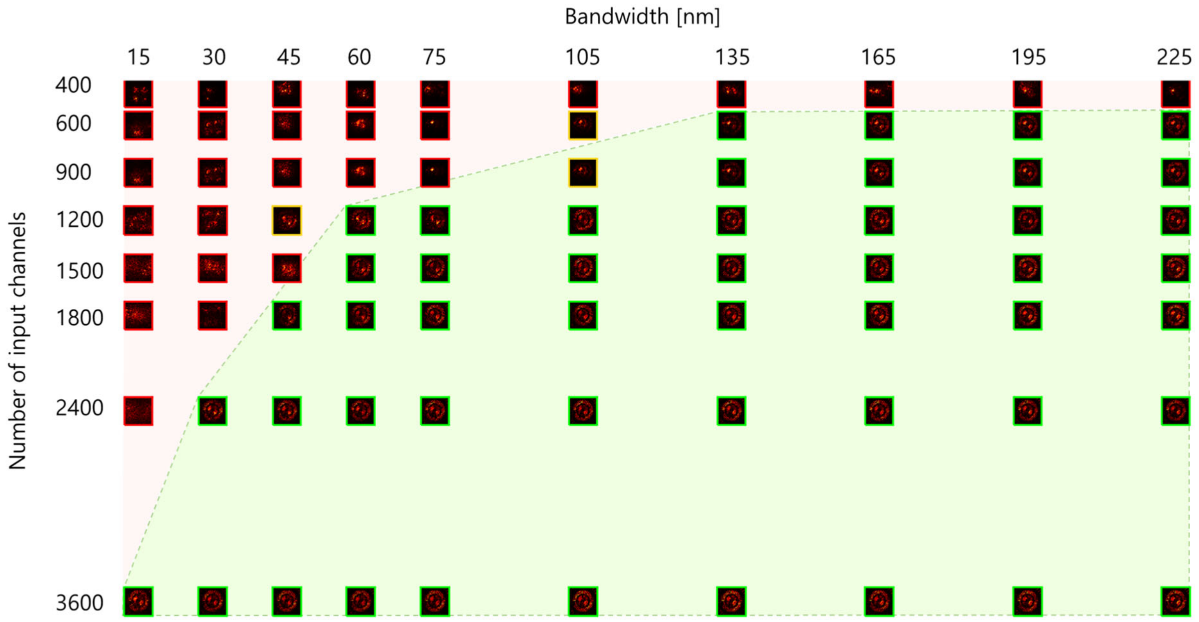

Figure S7. Image reconstruction results of VRM depending on N_λ and N_θ. The data in Fig. 4 of manuscript was analyzed for various bandwidths (or the number of wavelength channels N_λ) and the number of input angular channels N_θ. The green box indicates a successful reconstruction, the red box indicates a failed reconstruction, and the yellow box indicates a borderline image. Successful reconstructions are shaded in green while failed reconstructions are shaded in red.

The imaging depth limit of this sample can be deduced from the attenuation of single- and multiple-scattered waves depending on imaging depth. In the reflection-mode imaging, the intensity of single-scattered waves S and that of multiple-scattered waves M can be described as $S(z) = e^{-2z/l_\mathrm{s}}$, and $M(z) = e^{-2z/l_\mathrm{m}}$, respectively. Here l_s corresponds to the mean free path of single-scattered waves, and l_m represents the attenuation length of multiple scattering. l_m depends on various factors such as the numerical aperture, field of view, and the types of gating operations. It is longer than l_s in general, e.g. typically, $l_\mathrm{m}{\sim}1.5\, l_\mathrm{s}$ when the temporal gating is applied. The SMR ($\equiv S/M$) can be described as $\mathrm{SMR}_0(z) = e^{-\frac{2z}{l'}}$, where $l' = \frac{l_\mathrm{m} l_\mathrm{s}}{l_\mathrm{m} - l_\mathrm{s}}$ corresponds to the attenuation length of SMR. Let us set the original imaging depth $z_0 (= 9.4\, l_s)$ as $C_\mathrm{rel}(z_0) \approx \mathrm{SMR}_0(z_0)\zeta\sqrt{600} = C_\mathrm{th}$. If we increase N_θ to 5500, the achievable imaging depth z_d is given by the condition, $C_\mathrm{rel}(z_d) \approx \mathrm{SMR}_0(z_d)\zeta\sqrt{5500} = C_\mathrm{th}$.

Therefore, imaging depth limit of this sample can be calculated as

$$z_d = z_0 + \frac{l_\mathrm{m} l_\mathrm{s}}{l_\mathrm{m} - l_\mathrm{s}} \frac{\ln\left(\sqrt{5500/600}\right)}{2} \sim 9.4\ l_s + 1.7\ l_s = 11.1\ l_s.$$

VIII. Typical reflectance images of TiO_2 particles

Here we present the comparison of the conventional Full-Field (FF) OCT image and our VRM image for the TiO_2 particles near the surface to show that Figs. 6i-k are typical object images of the TiO_2 particles. Note that VRM image is sharper than FF-OCT image because VRM adds confocal axial gating to FF-OCT.

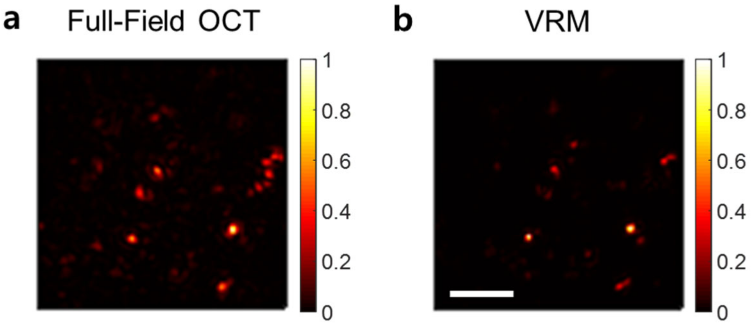

Figure S8. Typical reflectance images of TiO_2 particles near the surface of the scattering medium. a, Full-Field (FF) OCT image. **b,** VRM image. Scale bar, 10 μm. Color bar, normalized intensity by the maximum intensity in each image.

IX. List of symbols used in the main text

$E(\mathbf{r}; \boldsymbol{\theta}_\mathrm{in}, \lambda)$	Electric field in position basis
$\boldsymbol{\theta}_\mathrm{in}$	Incident angle
λ	Wavelength
$\Delta\lambda_\mathrm{F}$	Full spectral bandwidth
N_λ	The number of sampled wavelengths
$\tilde{E}(\mathbf{k}; \boldsymbol{\theta}_\mathrm{in}, \lambda)$	Spatial frequency spectrum of the reflected wave
$\mathbf{k}_\mathrm{in}$	Input wavevector
$\mathbf{k}$	Output wavevector
α	The numerical aperture of the objective lens
$\tilde{E}_\mathrm{S}(\mathbf{k}; \mathbf{k}_\mathrm{in}, \lambda)$	Single-scattered wave
$\tilde{E}_\mathrm{M}(\mathbf{k}; \mathbf{k}_\mathrm{in}, \lambda)$	Multiple-scattered waves

$O(\mathbf{r}, z_t)$	Object function
$\tilde{O}(\mathbf{k}, z)$	Spatial frequency spectrum of the object function
l_s	Scattering mean free path
$k_z^{\mathrm{in}}(\lambda) = \sqrt{k_0(\lambda)^2 - \lvert\mathbf{k}_{\mathrm{in}}\rvert^2}$	z-components of the wavevectors of the incident waves
$k_z(\lambda) = \sqrt{k_0(\lambda)^2 - \lvert\mathbf{k}\rvert^2}$	z-components of the wavevectors of the reflected waves
$k_0(\lambda) = 2\pi/\lambda$	Free-space wavenumber
$\tilde{E}_{\mathrm{cg}}(\mathbf{k}, z_t; \mathbf{k}_{\mathrm{in}})$	Coherence-gated field for a target depth z_t
$\tilde{E}_{\mathrm{ccg}}(\mathbf{K}, z_t)$	Confocal and coherence-gated field
$\mathbf{K} = \mathbf{k} - \mathbf{k}_{\mathrm{in}}$	Momentum difference of input and output wavevector
N_λ	The number of wavelengths scanned by the light source
$N(\mathbf{K})$	The number of $\mathbf{k}_{\mathrm{in}}$ and $\mathbf{k}$ pairs that meet the relation, $\mathbf{K} = \mathbf{k} - \mathbf{k}_{\mathrm{in}}$
$\phi_{\mathrm{in}}(\mathbf{k}_{\mathrm{in}}, \lambda)$	Input spectro-angular dispersion
$\phi_{\mathrm{o}}(\mathbf{k}, \lambda)$	output spectro-angular dispersion
λ_c	Center wavelength
$\langle 0 \rangle_{\mathbf{k}}$	The summation of the elements within the bracket with respect to **k**
$C_{\mathrm{rel}} \approx \frac{S}{M}\zeta\sqrt{N}$	Fidelity metric for finding dispersion correction
S	Average intensities of single- scattered waves at each detection channel
M	Average intensities of multiple- scattered waves at each detection channel
N	The total number of elements involved in the cross-correlation of pupil functions
ζ	The complexity of both input and output angular dispersions
$\tilde{E}_{\mathrm{MD}}(\mathbf{k}; \mathbf{k}_{\mathrm{in}})$	Depth merged multiple coherence-gated fields
N_z	The number of independent merged depths in the volumetric dispersion correction approach

Supplementary Table 3. List of symbols used in the main text
